# Expansion and Functional Divergence of AP2 Group Genes in Spermatophytes Determined by Molecular Evolution and *Arabidopsis* Mutant Analysis

**DOI:** 10.3389/fpls.2016.01383

**Published:** 2016-09-20

**Authors:** Pengkai Wang, Tielong Cheng, Mengzhu Lu, Guangxin Liu, Meiping Li, Jisen Shi, Ye Lu, Thomas Laux, Jinhui Chen

**Affiliations:** ^1^Ministry of Education, Key Laboratory of Forest Genetics and Biotechnology, Nanjing Forestry UniversityNanjing, China; ^2^Suzhou Polytechnic Institute of AgricultureSuzhou, China; ^3^Laboratory of Biotechnology, Chinese Academy of ForestryBeijing, China; ^4^Institute of Biology III, University of FreiburgFreiburg, Germany

**Keywords:** AP2 group gene, spermatophyte, phyllogeny, selective pressures, arabidopsis mutants, functional divergence

## Abstract

The *APETALA2* (*AP2*) genes represent the AP2 group within a large group of DNA-binding proteins called AP2/EREBP. The *AP2* gene is functional and necessary for flower development, stem cell maintenance, and seed development, whereas the other members of AP2 group redundantly affect flowering time. Here we study the phylogeny of AP2 group genes in spermatophytes. Spermatophyte AP2 group genes can be classified into AP2 and TOE types, six clades, and we found that the AP2 group homologs in gymnosperms belong to the AP2 type, whereas TOE types are absent, which indicates the AP2 type gene are more ancient and TOE type was split out of AP2 type and losing the major function. In Brassicaceae, the expansion of AP2 and TOE type lead to the gene number of AP2 group were up to six. Purifying selection appears to have been the primary driving force of spermatophyte AP2 group evolution, although positive selection occurred in the AP2 clade. The transition from exon to intron of *AtAP2* in *Arabidopsis* mutant leads to the loss of gene function and the same situation was found in *AtTOE2*. Combining this evolutionary analysis and published research, the results suggest that typical AP2 group genes may first appear in gymnosperms and diverged in angiosperms, following expansion of group members and functional differentiation. In angiosperms, *AP2* genes (AP2 clade) inherited key functions from ancestors and other genes of AP2 group lost most function but just remained flowering time controlling in gene formation. In this study, the phylogenies of AP2 group genes in spermatophytes was analyzed, which supported the evidence for the research of gene functional evolution of AP2 group.

## Introduction

The genes in AP2/ERF family can be divided into subfamilies according to the number of AP2/ERF domains. There is a single AP2/ERF domain in each member of the EREBP subfamily, which function in the signal transduction pathways of stress responses and cambial tissue development (Mizoi et al., 2012; Licausi et al., 2013). The members of the AP2 subfamily contain two AP2/ERF domains is further classified into two monophyletic groups: the AP2 group and the AINTEGUMENTA (ANT group, Shigyo et al., 2006). We selected the AP2 group genes for our study because they played key roles in the reproductive and vegetative organs development (Ohto et al., 2005; Huijser and Schmid, 2011).

There are seven conservative domains in a typical AP2 group gene, including one ethylene-responsive element binding factors (ERF) -associated amphiphilic repression motif (EAR) (Kagale et al., 2010) or EAR-like domain, a nuclear localization signal (NLS) domain, two AP2 (AP2-R1 and AP2-R2) domain (Kim et al., 2006), a linkage domain (connecting the AP2-R1 with R2), another EAR domain, and a *MIR172* target site (Image 1). However, not all AP2 group genes contain two typical complete AP2 domains. For example, there are six members in the AP2 gene group in *Arabidopsis, TARGET OF EARLY ACTIVATION TAGGED 1-3* (*TOE1-3*), *AP2, SCHLAFMUTZE* (*SMZ*), and SCHNARCHZAPFEN (*SNZ*). Among these six *Arabidopsis* genes, *AP2, TOE3*, and *TOE1* contain both complete AP2 domains (AP2-R1 and R2 domains) but there is only one typical AP2 domain (AP2-R1 domain) in *TOE2, SMZ*, and *SNZ* (Image 1). The AP2-R2 domain in these three genes are not the same as *AP2, TOE3*, and *TOE1*.

In *Arabidopsis*, AP2 regulates floral development, (Jofuku et al., 1994), stem cell maintenance, (Wurschum et al., 2005), seed developmenta, whereas the remaining five genes (*TOE1-3, SMZ*, and *SNZ*) act redundantly as flowering repressors (Zhu and Helliwell, 2011). mRNA abundance and translation of *TOE1-2, AP2, SMZ*, and *SNZ* in *Arabidopsis* are regulated by *miR172*, which is also important for regulating phase transition and determining floral organ identity in monocotyledons (Nair et al., 2010; Zhu and Helliwell, 2011). *TOE3* most likely acts redundantly with *TOE1* and *TOE2* to repress flowering. A good candidate for such a repressor is SMZ, which was originally identified in an activation-tagging screen because of its dominant late-flowering phenotype. Additionally, *SNZ*, a paralog of *SMZ*, represses flowering when expressed at high levels. Several other known regulators of flowering time have been identified as *SMZ* targets. Among them are *SMZ* itself, *SNZ, AP2*, and *TOE3*, suggesting complex feedback regulation among AP2 group members.

Several studies have examined the origin, phylogeny and evolution of the AP2/ERF family and the AP2 subfamily (Magnani et al., 2004; Kim et al., 2006; Shigyo et al., 2006). With the development of high-throughput DNA sequencing techniques, increasing information has become available in recent years for the of AP2/ERF gene family based on genomic data from species such as rice, apple, peach, *Hevea brasiliensis, Prunus mume, Vitis vinifera, Populus trichocarpa*, Chinese cabbage, and others (Zhuang et al., 2008; Licausi et al., 2010; Sharoni et al., 2011; Zhang et al., 2012; Du et al., 2013; Duan et al., 2013; Song et al., 2013). However, these studies have mainly focused on the identification, classification and expression of AP2/ERF family genes. To date, there have been no studies describing the molecular evolutionary history and the structural characterization of the AP2 group in spermatophytes. As such, an evolutionary and structural analysis of this group may provide both a reference for further functional studies and evidence for gene functional diversification.

Here, we examined 105 spermatophyte AP2 group genes from 56 spermatophytes by phylogenetic analyses, comparing whole gene sequences, homeodomains, and other motifs. The results revealed that, in general, the spermatophyte AP2 group experienced background purifying selection throughout evolution. However, analyses also showed that the AP2 group genes have also undergone positive selection, despite little evidence for positive pressure on these genes. In particular, the evolutionary relationships among members of the AP2 group were apparent from the divergence between the TOE and AP2 type. By analyzing the expression patterns, functional data and phylogenetic relationships among *AP2* genes, we reveal rules concerning the formation of new genes in the AP2 group and identified the pathway of functional evolution. We also find evidenc that the AP2 function in maintaining the stem-cell niche is to be conserved in spermatophytes.

## Results

### The orthologs of AP2 group genes from spermatophytes differ and can be classified into two types and six clades

The composition of AP2 group orthologs differs among spermatophyte species. It is well known that the *Arabidopsis* AP2 group has six members, namely *AtAP2, AtTOE1–3, AtSMZ*, and *AtSNZ*. In fact, blast analysis of spermatophyte AP2 group gene sequences revealed that only some species in Brassicaceae contain five or six AP2 group members. Orthologs of *AtTOE2, AtTOE3, AtSMZ*, and *AtSNZ* were not found in the other species included in our study. Although the AP2-R2 domain in the *AtTOE1* orthologs of some species is not complete, these genes still clustered together to form the TOE1 clade in the prephylogenetic analysis. Therefore, the orthologs of *AtTOE2, AtTOE3, AtSMZ*, and *AtSNZ* are only been identified from Brassicaceae (Data sheet 1).

**Figure 1:**
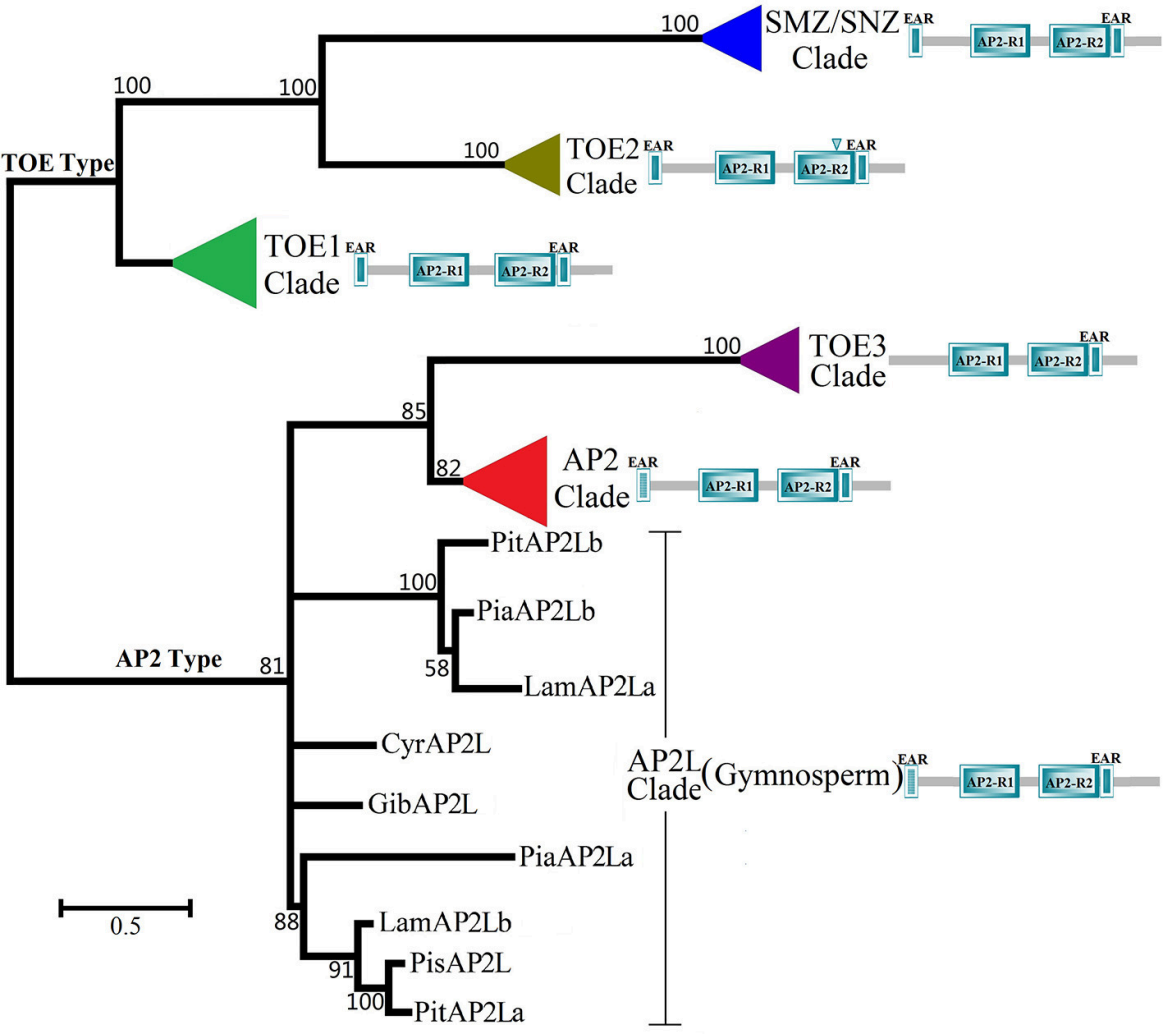
**Maximum-likelihood Phylogenetic Tree of AP2 Group Protein in Spermatophyte (simplified phylogenetic tree)**. The ML tree was constructed based on the whole protein sequences of spermatophyte AP2 Group gene using MEGA6.0 with 1000 bootstrap replications and Jones-Taylor-Thornton (JTT) + Gamma Distributed model with Invariant sites model (Discrete Gamma Categories = 5). There were 105 spermatophyte AP2 group genes, which were searched from 23 families, 56 spermatophytes. The different color triangles represent different clades except six gymnosperms (red, AP2 clades; green, TOE1 clade; olive, TOE2 clade; purple, TOE3 clade; blue, SMZ/SNZ clade). The detailed phylogenetic tree of every clade was shown in Image 2. The scale bar indicates the branch length that corresponds to 0.5 substitutions per site. The species and accession numbers are listed in Data sheet 1. The abbreviations used are as follows: *Pit, Pinus thunbergii*; *Pia, Picea abies*; *Lam, Larix* × *marschlinsii*; *Cyr, Cycas revoluta*; *Gib, Ginkgo biloba*; *Pi*s, *Picea sitchensis*.

All predicted spermatophyte AP2 group protein sequences (105, Data sheet 1) were retrieved from the plant genome (Phytozome and NCBI) and protein databases (NCBI) and used to construct a maximum-likelihood phylogenetic tree (Figure 1 and Image 2). According to the simplified phylogenetic tree (Figure 1) of spermatophyte AP2 group, all genes were categorized as two types: the AP2 type, which included the three clades TOE3, AP2-like and AP2, and the TOE1 type, which included the three clades TOE1, TOE2, SMZ/SNZ. The results of the phylogenetic analysis were consistent with those of the sequence search. For each ortholog, most of the spermatophyte sequences clustered together to form an independent clade, except in gymnosperms. The genes *AP2, TOE1, TOE2, SMZ*/*SNZ*, and *AP2L* from gymnosperms *Cycas revoluta* (*CyrAP2L*), *Ginkgo biloba* (*GibAP2L*), *Picea sitchensis* (*PisAP2L*), *Larix* × *marschlinsii* (*LamAP2La, b*), *Picea abies* (*PiaAP2La, b*), and *Pinus thunbergii* (*PitAP2La, b*) are well clustered into form six independent clades (Figure 1, bootstrap value >80%). *AP2L* sequences from gymnosperms were obtained from the NCBI database and clustered together with the AP2 and TOE3 clades to form a larger group, which implied that *AP2* genes might be relatively ancient in the AP2 group. The two sub-branches of Pinaceae in the AP2L cluster c adequately reflected the duplication of AP2 genes in gymnosperms. In each clade, most of the sequences from species within a single family or order clustered together well to form an independent group (Image 2, bootstrap value >69%). These results indicated that most of these sequences are specific at the family level. Intriguingly, in the branches of AP2 and TOE1, the phylogenetic trees are very similar to the structure of the Angiosperm Phylogeny Group system, which classifies basal angiosperms, monocots, and dicotyledons into three independent groups. In the AP2 clade, the dicots such as *Ricinus communis, Manihot esculenta, Jatropha curcas, Populus trichocarpa, Carica papaya, Vitis vinifera*, and *Betula platyphylla* with unisexual flowers formed a branch with lower bootstrap value (bootstrap value = 25). However, the branch of unisexual flower didn't appear in the TOE1 clade.

### The distribution of homeodomains varies significantly in different clades and types

There are seven common homeodomains in the typical AP2 group genes according the analytical results of MEME and Pfam (Bailey et al., 2009; Punta et al., 2011): the first EAR domain (DLNxxP or LxLxL), NLS domain, AP2-R1 domain, linkage domain, AP2-R2 domain, the second EAR domain (LxLxL), and miRNA172 target site. These homeodomains except miRNA172 target site are conserved in amino acid sequences. NLS domain, AP2-R1 domain, linkage domain, the second EAR domain and miRNA172 target site have greater sequence similarity than the first EAR domain and AP2-R2 domain.

Notably, the EAR domains differed among the clades. In the TOE3 clade (Image 2), there is no first EAR domain. Likewise, PtAP2, AdAP2, EgAP2, StAP2, MdTOE1, AtTOE1, GpTOE1, NhTOE1, and orthologs from the Rutaceae family also do not contain the first EAR domain. Interestingly, both EAR domains are missing in TOE1 orthologs from the Poaceae family. *LamAP2La, PiaAP2La*, and *PisAP2L*, three complete protein-coding genes in the AP2L clade, contain two EAR motifs. Because some N-terminal amino acid sequences of the AP2L clade were incomplete, it was not clear whether the first EAR domain exists in all AP2L clade members from gymnosperms. The EAR domains are of two types, namely DLNxxP and LxLxL. The amino acid sequence of the first EAR domain in the AP2 type (including LamAP2La, PiaAP2La, and PisAP2L) was DLNxxP, but the sequence in the TOE1 type was LxLxL, which is the same as the second EAR domain (LxLxL). Outside of the EAR domains, differences in the AP2-R2 domain were identified among AP2 group proteins. There was an incomplete AP2-R2 domain in the amino acid sequence of the TOE2 clade lacking a 15-residue insertion (Image 1). There are significant amino-acid sequence differences between the typical AP2-R2 domain and the SMZ/SNZ clade. The TOE2 clade has a closer phylogenetic relationship to the SMZ/SNZ clade, forming a TOE type with the TOE1 clade, indicating a common ancestry separate from the AP2 type.

### The distribution of motifs reflects differences and phylogenetic relationships across clades and species

**Figure 2:**
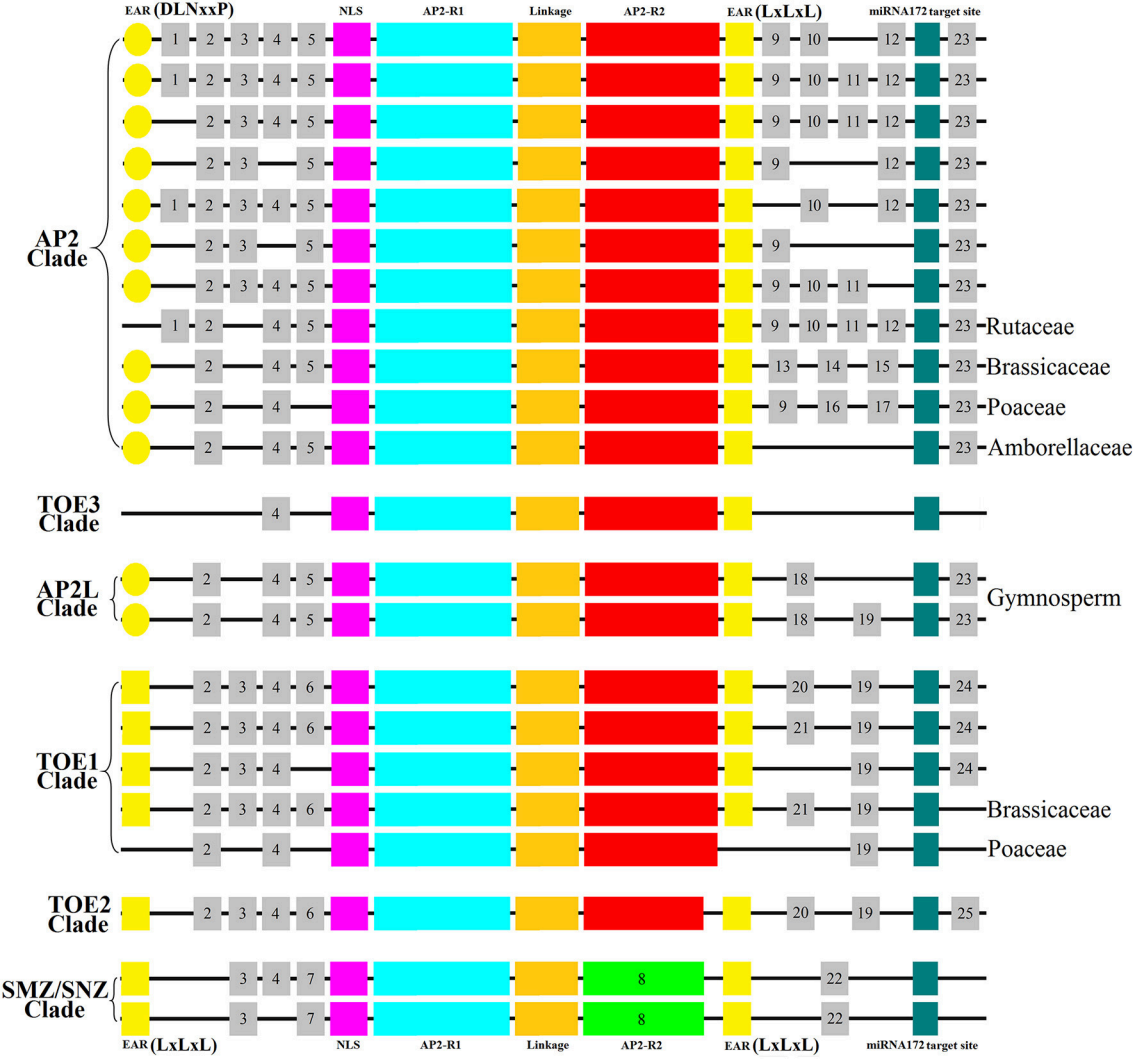
**Distribution of Homeodomains and Motifs of AP2 Group in Spermatophyte**. A schematic representation of motifs obtained using MEME within the sequences is displayed. The homeodomains (EAR domain, NLS domain, AP2-R1 domain, linkage domain; AP2-R2 domain, miRNA172 target site) were showed by the same colors with Image 1. The different motifs were indicated by using numbers.

Motifs of the AP2 group were investigated, and 25 motifs including the AP2-R2 domain of the SMZ/SNZ clade, i.e., Motif 8, were then characterized relative to the homeodomains (Figure 2). Most of these motifs were located upstream of the NLS (7/25) or downstream of the second EAR domain (14/25). Only one motif was identified at the C-terminus (i.e., the downstream of the miR172 target site) of most AP2- and TOE-type proteins, and no C-terminal motif was seen in the proteins of clades TOE3 and SMZ/SNZ and TOE1 proteins of the Brassicaceae and Poaceae families (Figure 2). We also noted that most AP2 clade proteins contained more motifs (15/25), but there was only one motif (Motif 4) in TOE3 clade proteins. The differences in numbers of motifs were seen mostly in one region, i.e., between the second EAR motif and the miR172 target site. Most AP2 clade proteins had three or four motifs, whereas other proteins had only one or two. The different clades and species could be characterized based on the distribution of motifs. Every clade contained its own unique motifs: AP2 clade, Motifs 1 and 9–17; AP2L clade, Motif 18; TOE1 clade, Motifs 21 and 24; TOE2 clade, Motif 25; SMZ/SNZ clade, Motifs 7, 8, and 22). This indicated that the motifs might be related to the functional divergence of the AP2 group (Figure 2). Motif 4 was shared by almost all AP2 group proteins (Figure 2).

Interestingly, the distribution of motifs revealed a phylogenetic relationship among AP2 group proteins that agreed with the results of the phylogenetic analysis by MEGA (Figure 1). In the AP2L clade, all sequences were from gymnosperms and contained five or six motifs (Motifs 2, 4, 5, 18, 19, and 23), suggesting that these motifs are more primitive. Notably, Motifs 5 and 23 are specific to clades AP2 and AP2L, and Motif 19 is also specific to clades TOE1 and TOE2, which may reflect the phylogenetic relationship between AP2 group members from clade AP2L and clades AP2 and TOE1. The proteins in the AP2 clade from Brassicaceae and Poaceae contain unique motifs, i.e., Motifs 13–15 in Brassicaceae and Motifs 16 and 17 in Poaceae, forming two separate branches. By contrast, Brassicaceae and Poaceae TOE1 proteins lacked unique motifs, suggesting that *AP2* evolved faster than *TOE1*. Another observation supporting this view is that Motif 19 is present in clades AP2L and TOE1 but not in clade AP2.

### Purifying selection was the main driving force in the evolution of spermatophyte AP2 group genes, but positive selection still occurred, mostly in clade AP2

**Figure 3:**
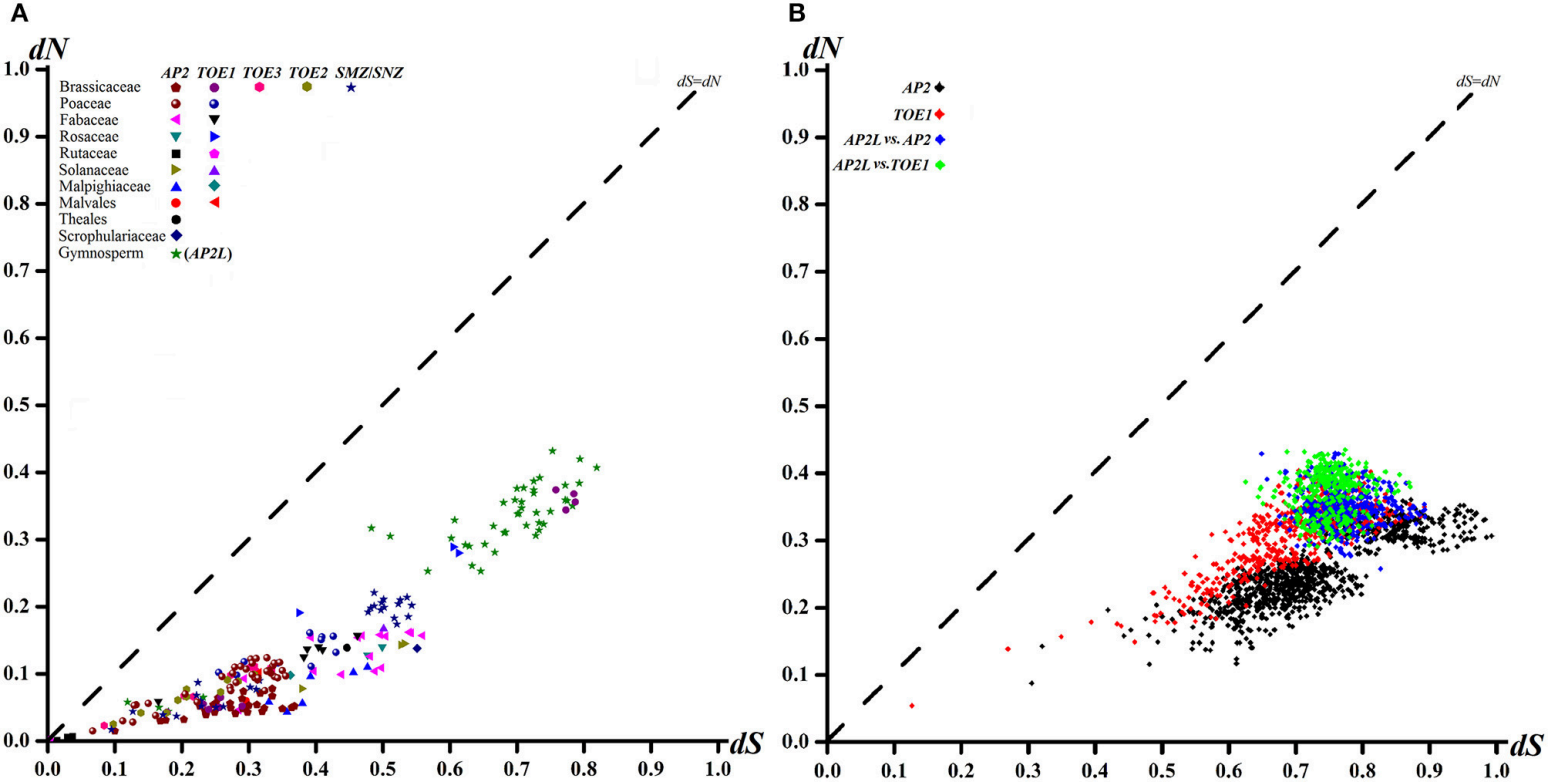
**Pairwise Comparison Plots of ***dN*** and ***dS*** Values for each family (order) AP2 group genes (A) and different clades (B)**.

One criterion for assessing the type of selective pressure at the protein level is to calculate ω, i.e., *dN*/*dS*, for protein-coding genes (Seo et al., 2004; Kryazhimskiy and Plotkin, 2008). The *dN*/*dS* ratio ω provides a criterion for assessing selective pressure at the protein level (Zhang et al., 2006). The ω-values of >1, 1 and < 1 imply positive selection, neutral evolution and purifying selection, respectively. In the graph of the AP2 group sequences, most of the points fell between the *dS* axis and the diagonal, indicating *dN* < *dS* (Figure 3) and suggesting that purifying selection dominated the selection process during evolution. Similar ω-values were obtained for each family (order) (Figure 3B), which also contained the different clades and the comparison between them (*p* < 0.01, *Z-test*, Figure 3B). Calculation of the ratio of nucleotide substitutions in a one-by-one comparison of *dN* to *dS* for individual AP2 group genes within families (orders) (Figure 3A) and clades (Figure 3B) provided further evidence for purifying selection.

**Figure 4:**
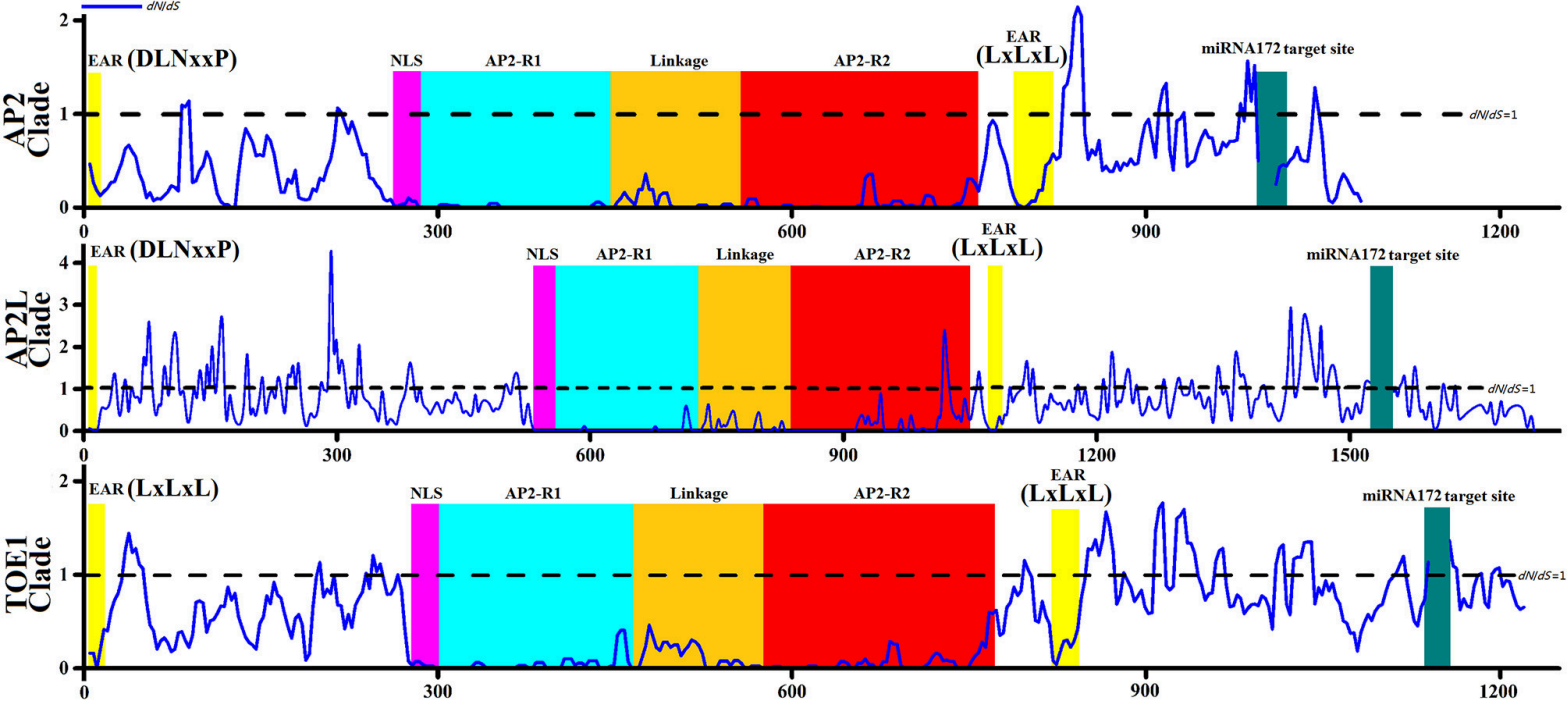
**Sliding-window Analyses of ***dN***/***dS*** among Three Clades Genes (AP2 clade,AP2L clade, and TOE1 clade)**. The sliding-window analyses were performed using a 30-bp sliding window moving in steps of 3-bp. Numbers on the x-axis represent the sequence positions followed below by the domain map of genes. The homeodomains (EAR domain, NLS domain, AP2-R1 domain, linkage domain; AP2-R2 domain, miRNA172 target site) were showed by the same colors with Image 1. The straight lines shown in the figure represent *dN* = *dS*.

The *dN* of the TOE1 clade was higher than that of the AP2 clade (Figure 3B), implying that more amino acids changes accumulated in TOE1 during evolution, similar to the results of the sliding-window analyses of ω among clades AP2, AP2L, and TOE1 (Figure 4). The sliding-window ω-tests on clades AP2, AP2L, and TOE1 (Figure 4) showed similar ω curves in the different clade lineages. The ω-values of the seven common homeodomains (the two EARs, NLS, AP2-R1, linkage, and AP2-R2 domains and the miR172 target site) in the three lineages were almost all much less than 1 except for the end of the AP2-R2 domain in AP2L, which further suggested the functional importance of the common homeodomains in AP2 group proteins. The ω-values are not shown for the miR172 target site because *dN* was zero. The peaks above the line that marks *dN* = *dS* in Figure 4 suggest the existence of positive selection, primarily on either side of the two AP2 domains, especially downstream of domain AP2-R2. There were more *dN* > *dS* peaks in the graph of the AP2L clade because fewer AP2 ortholog sequences are available for gymnosperms (only eight sequences) for analysis of evolutionary pressure, but the distribution trend of positive-selection peaks agreed with that of clades AP2 and TOE1. Compared with clade AP2, TOE1 contained more positive-selection peaks, implying that the purifying selective pressure on the TOE1 clade was relatively weak and enabled more nonsynonymous substitutions to be retained. Potentially, the ω-value differences of different regions are related to functional divergence in AP2 group genes.

To examine if ω varied among branches of each clade (Figure 1), the free-ratio and one-ratio models in the Codeml program of PAML 4.2 were chosen and used to detect selective pressure acting on some branches (Data sheet 2). The values of ω for these AP2 group genes were similar (0.2083–0.2897) and substantially less than 1. However, the free-ratio model fit the data better than the one-ratio model for the protein-coding sequences from clades AP2, AP2L, and TOE1, suggesting that the genes from these three clades possibly experienced different selective pressures. Conversely, when coding sites in genes of clades TOE3, TOE2, and SMZ/SNZ were analyzed, the codon-substitution free-ratio model, which allows for different ω-values among the branches, did not fit the data any better than the one-ratio model, which assumes a single mean ω-value for the branches. The primary reason for this result was that the genes in clades TOE3, TOE2, and SMZ/SNZ were all from Brassicaceae. All AP2 group genes were analyzed by the free-ratio and one-ratio models, and the results also suggested that ω varied among branches. Therefore, genes of different clades experienced different selective pressures.

Six codon substitution models, namely M0 (one-ratio), M1a (nearly neutral), M2a (positive selection), M3 (discrete), M7 (beta), and M8 (beta and ω), were implemented in PAML 4.2 to analyze the positive selection and identify positively selected sites in all AP2 group genes. The likelihood values and parameter estimates of all AP2 group gene sequences from the six models applied in the Codeml program are listed in Table 1. The average ω-values in the six models ranged from 0.2794 to 0.5952, providing evidence for purifying selection. Although the average ω was 0.2794 for all sites of the AP2 group genes by the M0 model, this model was rejected as a result of the low-likelihood value (−87829.48516) and the LRT statistic (2 delta lambda statistic, 2Δ*l*, Table 1). No positively selected sites were identified by the M3 model because ω < 1, but two models (M2a and M8) allowed for positive selection, indicated by 38 and 17 positively selected sites with ω > 1, respectively. Because of the overestimate of the number of actual positively selected sites (Anisimova et al., 2001, 2002), the results under model M3 were not used to identify positively selected sites. To reduce or avoid possible false-positive results, positively selected sites identified simultaneously by models M2a and M8 in Codeml were defined as positively selected. The LRT statistic demonstrated that the two selection models fitted the data significantly better than the null models without positive selection, supporting the view that certain amino acids in AP2 group proteins experienced strong positive selection. At the level of posterior probability >0.95, 22, and 9 sites in the AP2 group genes were identified as being under positive selection (ω > 1) by the selection models M2a and M8, respectively (Table 1). There were 15 sites with posterior probability >0.99 among the 22 positively selected sites in the M2a model and 7 among the 9 positively selected sites in the M8 model. All 9 positively selected sites detected by M8 were also identified by M2a at the level of posterior probability >0.99. The positively selected sites were mainly concentrated in two regions—upstream of the NLS and downstream of the second EAR domain. Both regions showed the corresponding positive-selection peaks in Figure 4. All of this evidence supports the existence of positive selection and positively selected sites in AP2 group genes during spermatophyte evolution.

**Table 1:** **Likelihood values and parameter estimates for AP2 group genes sequences in Spermatophyte**.

**Model code**	**InL**	***dN*/*dS***	**Estimates of parameters**	**2*Δl***	**Positive selection**
M0 (one-ratio)	−87829.4852	0.2794	ω = 0.2794		None
M3 (discrete)	−81346.4866	0.3541	*p*_0_ = 0.4298, *p*_1_ = 0.2336 (*p*_2_ = 0.3365), ω_0_ = 0.0223, ω_1_ = 0.3017 ω_2_ = 0.8143	12965.9972 (*P* = 0.0000)	None
M1a (NearlyNeutral)	−82440.5178	0.5171	*p*_0_ = 0.5144 (*p*_1_ = 0.4856)		Not allowed
M2a (PositiveSelection)	−82283.7557	0.5952	*p*_0_ = 0.5014, *p*_1_ = 0.4197 (*p*_2_ = 0.0789), ω_0_ = 0.0594, ω_1_ = 1.0000 ω_2_ = 1.8466	313.5242 (*P* = 0.0000)	44D **54G** 57V **74G 75S 76S 77A 78G 79K 80A** 81T **82N 83V** 276H **279Q** 284R **286N 287Q 289Q 290Q** 291L 353T
M7 (beta)	−81006.4547	0.3169	*p* = 0.2595, *q* = 0.5591	87.1449 (*P* = 0.0000)	Not allowed
M8 (beta & ω)	−80962.8822	0.3433	*p*_0_ = 0.9578 (*p*_1_ = 0.0422),		**74G 75S 76S 77A** 79K **80A 82N** 287Q **289Q**
			*p* = 0.3013, *q* = 0.727, ω = 1.5014		

*lnL: the log-likelihood difference between the two models; 2Δl: twice the log-likelihood difference between the two models. The values in parentheses represent the significant level of 0.01 with a χ2 distribution at d.f. = 4 (M0 vs. M3) or 2 (M1a vs. M2a and M7 vs. M8). The amino acid sequence of AtAP2 was used as the sequence reference, and positive selected sites were identified with posterior probability p > 0.95. In boldface, p > 0.99*.

The likelihood values and parameters of the three main clade branches (AP2, AP2L, and TOE1) of AP2 group proteins were estimated by the six models to detect whether there was positive selection in clades AP2, AP2L, and TOE1 (Table 2). Positive selection was only detected in clade AP2 by the two positive selection models (M2a and M8), and no such positively selected sites were discovered in AP2L and TOE1. The positively selected sites in clade AP2 were not identified by the M3 (discrete) model because ω was < 1, which was similar to the results for all AP2 genes. Of the sites with posterior probability >0.95 in AP2 clade proteins, six and four sites were identified to be under positive selection (ω > 1) by selection models M2a and M8, respectively, and the number of positively selective sites was four and two in M2a and M8, respectively, at posterior probability >0.99. The positively selected sites of clade AP2 were mainly concentrated upstream of the miR172 target site, and there were also corresponding positive-selection peaks in Figure 4. The parameter estimates from the six models were quite similar between clades AP2L and TOE1. The parameters of the M2a model for AP2L and TOE1 revealed a lack of positive selection in the two clades because ω = 1. The LRT statistic demonstrated that the M8 model did not fit the data significantly better than the M7 model without positive selection, suggesting that no amino acid sites in AP2L and TOE1 underwent positive selection. Therefore, the analysis of selective pressure of the three main clade branches of spermatophyte AP2 group genes indicated that clades AP2L and TOE1 experienced similar adaptive evolutionary mechanisms and only the AP2 clade underwent positive selection. Because *TOE2, TOE3, SMZ*, and *SNZ* are only found in Brassicaceae, the analysis of positive selection of Brassicaceae AP2 group genes and five branches (*AP2, TOE1, TOE2, TOE3*, and SMZ/SNZ) was performed (Data sheet 3), with the results revealing a lack of positive selection in the AP2 group genes of Brassicaceae, which indicates that the expansion of the number of AP2 group genes in Brassicaceae was not caused by positive selection.

**Table 2:** **Likelihood Values and Parameter Estimates for AP2, AP2L, and TOE1 Clades Genes Sequences**.

**Clade**	**Model code**	**InL**	***dN*/*dS***	**Estimates of parameters**	**2*Δl***	**Positive selection**
AP2	M0 (one-ratio)	−32964.4428	0.2083	ω = 0.2083		None
	M3 (discrete)	−30570.7018	0.2613	*p*0 = 0.5033, *p*1 = 0.3059 (*p*2 = 0.1908), ω0 = 0.0129, ω1 = 0.2785 ω2 = 0.8891	4787.48207 (*P* = 0.0000)	None
	M1a (NearlyNeutral)	−31071.0231	0.4177	*p*0 = 0.6109 (*p*1 = 0.3891)		Not allowed
	M2a (PositiveSelection)	−31036.8254	0.4636	*p*0 = 0.6053, *p*1 = 0.3574 (*p*2 = 0.0373), ω0 = 0.0466, ω1 = 1.0000 ω2 = 2.0876	68.395406 (*P* = 0.0000)	**298D 303D** 304S **306A 307G** 326S
	M7 (beta)	−30488.3157	0.2556	*p* = 0.2052, *q* = 0.5973	24.892428 (*P* = 0.0000)	Not allowed
	M8 (beta&ω)	−30475.8695	0.2654	*p*0 = 0.9713 (*p*1 = 0.0287)		**298D** 303D 306A **307G**
				*p* = 0.2195, *q* = 0.0466*, ω* = 1.5340		
AP2L	M0 (one-ratio)	−11930.6163	0.2897	ω = 0.2897		None
	M3 (discrete)	−11582.3172	0.3853	*p*0 = 0.2811, *p*1 = 0.5226 (*p*2 = 0.1963), ω0 = 0.0063, ω1 = 0.3192 ω2 = 1.1041	696.598213 (*P* = 0.0000)	24C 25S **50S** 66S 67M 68S 72P 111I **112V** 164A 172P 175A 356H 371D **391C** 435V **445P** 456R **462Q** 466S 467G 475D 522R 533M 534Q 538I 542P 543T 546A 547L
	M1a (NearlyNeutral)	−11675.8826	0.4763	*p*0 = 0.6021 (*p*1 = 0.3979)		Not allowed
	M2a (PositiveSelection)	−11675.8826	0.4763	*p*0 = 0.6021, *p*1 = 0.3028 (*p*2 = 0.0951), ω0 = 0.1302, ω1 = 1.0000 ω2 = 1.0000	NA	None
	M7 (beta)	−11599.3964	0.3641	*p* = 0.4323, *q* = 0.7545		Not allowed
	M8 (beta&ω)	−11597.7023	0.3896	*p*0 = 0.9625 (*p*1 = 0.0375) *p* = 0.4627, *q* = 0.9010, ω = 1.6976	3.388152 (*P* = 0.1838)	None
						
TOE1	M0 (one-ratio)	−31052.6234	0.2710	ω = 0.2710		None
	M3 (discrete)	−29048.0536	0.3427	*p*0 = 0.3819, *p*1 = 0.3179 (*p*2 = 0.3002), ω0 = 0.0129, ω1 = 0.2758 ω2 = 0.8330	4009.139619 (*P* = 0.0000)	None
	M1a (NearlyNeutral)	−29473.6938	0.5461	*p*0 = 0.4793 (*p*1 = 0.5207)		Not allowed
	M2a (PositiveSelection)	−29473.6938	0.5461	*p*0 = 0.4793, *p*1 = 0.0583 (*p*2 = 0.4624), ω0 = 0.0531, ω1 = 1.0000 ω2 = 1.0000	NA	None
	M7 (beta)	−29004.4021	0.3346	*p* = 0.2721, *q* = 0.5409		Not allowed
	M8 (beta&ω)	−29001.2895	0.3491	*p*0 = 0.9613 (*p*1 = 0.0387)		None
				*p* = 0.2834, *q* = 0.6361*, ω* = 1.3657	6.225359 (*P* = 0.0445)	

*lnL: the log-likelihood difference between the two models; 2Δl: twice the log-likelihood difference between the two models. The values in parentheses represent the significant level of 0.01 with a χ2 distribution at d.f. = 4 (M0 vs. M3) or 2 (M1a vs. M2a and M7 vs. M8). The amino acid sequences of AtAP2, PiaAP2La, and AtTOE1 were respectively used as the sequences reference. Positive selected sites in AP2 and AP2L clades were identified with posterior probability p > 0.95, In boldface, p > 0.99*.

### Certain amino acid residues in common homeodomains reflect the evolutionary relationship from *AP2L* to *AP2* and *TOE1*

**Figure 5:**
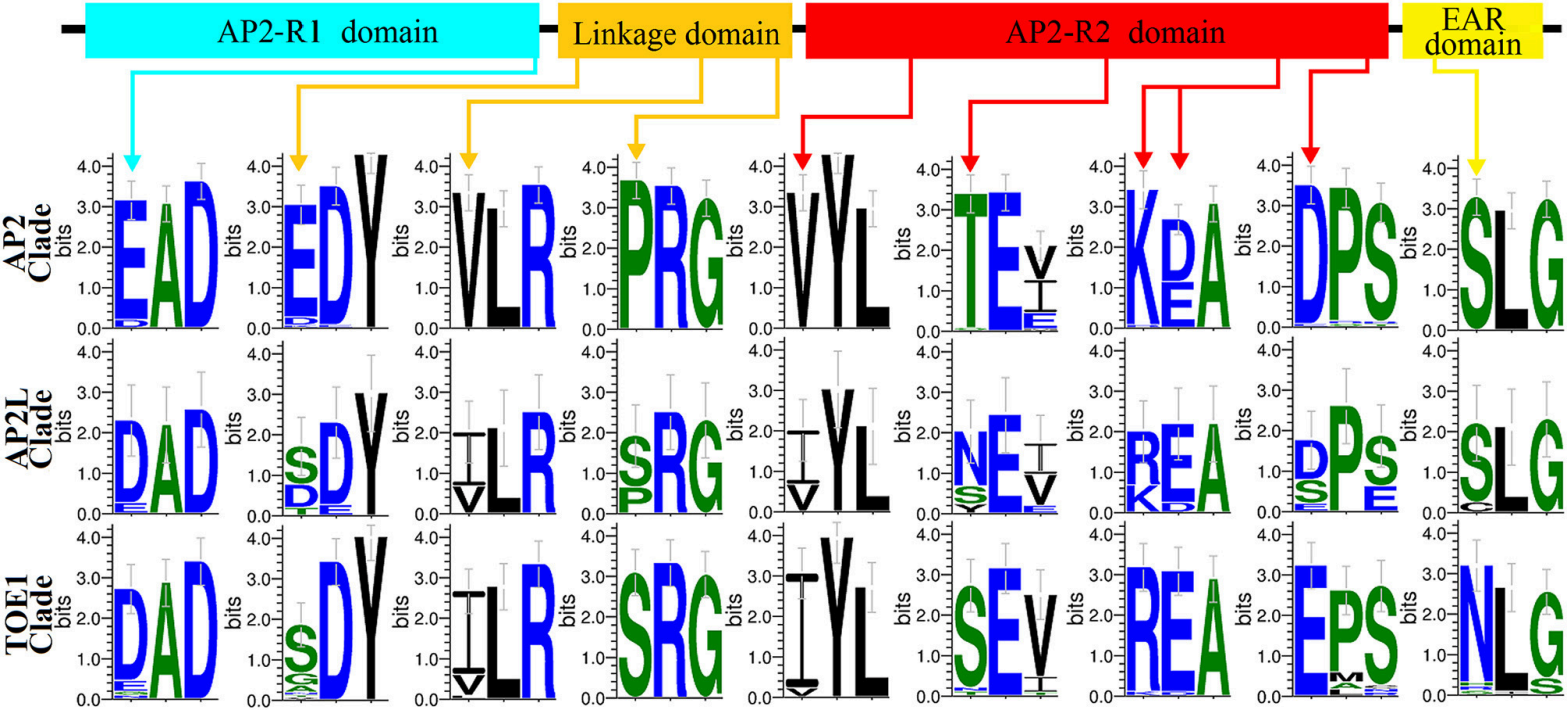
**Clade-specific sites in Homeodomains among Three Clades Genes (AP2 clade,AP2L clade, and TOE1 clade)**. Sequence logos of clade-specific sites in homeodomains identified in AP2Ls, AP2s, and TOE1s sequences. The arrows marked clade-specific sites and their position in homeodomains.

Both the distribution of motifs and the analysis of selective pressure suggested that *AP2L* may have diverged to yield the two structurally and functionally distinct genes *AP2* and *TOE1*. Specific motifs (Motifs 5, 19, and 23) of clades AP2 and TOE1 were also found in AP2L proteins, and AP2L and TOE1 experienced similar adaptive evolutionary processes. By comparing all AP2, AP2L, and TOE1 proteins, 10 amino acid sites that may reflect the evolutionary relationship in common homeodomains were identified (Figure 5), one in the AP2-R1 domain, three in the linkage domain, five in the AP2-R2 domain and one in the second EAR domain. These sites could be divided into three categories: AP2L having the same amino acids as TOE1 (three sites), AP2L having the same amino acids as AP2 (one site) and AP2L having two amino acids from AP2 and two from TOE1 (six sites). Only one site belonging to the second category suggested that *AP2* evolved faster than *TOE1*, in agreement with the results of the adaptive evolution analysis. The sites having two amino acids from AP2 and TOE1 also provided evidence that *AP2L* diverged into two structurally and functionally distinct genes, *AP2* and *TOE1* OR genes, *AP2* and *TOE1*, through structural and functional changes.

### Alignment of AP2 group gene homolog sequences from *A. thaliana* demonstrated the expanded mode of spermatophyte AP2 group

**Figure 6:**
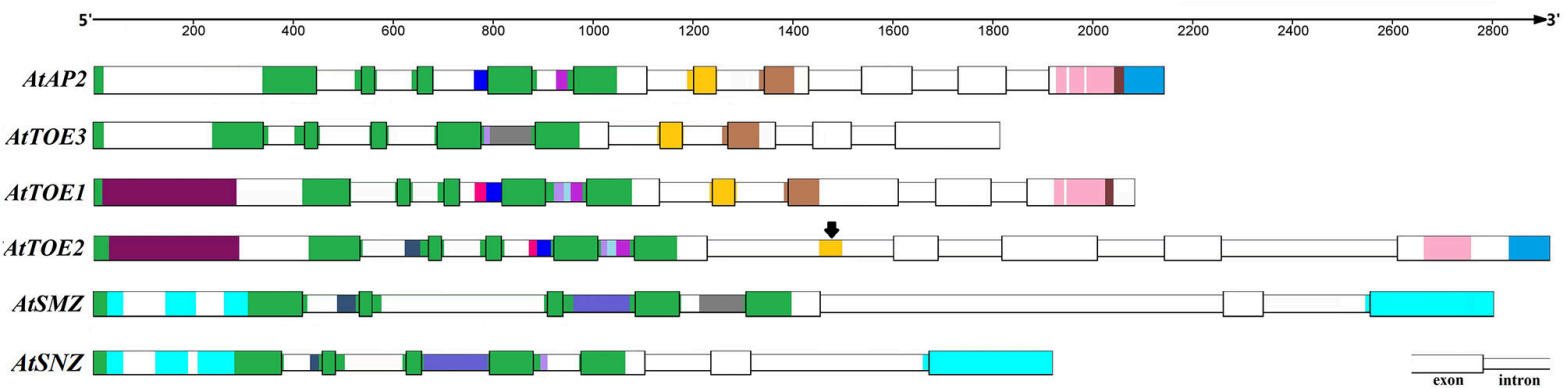
**Alignment of Genomic Sequences of AP2 Group Genes in ***A. thaliana*****. The sequence regions with same color in this figure have higher homology with each other. Black arrows means one intron region (gold) in *AtTOE2* has higher sequence similarity with the sixth exon in *AtAP2, AtTOE3*, and *AtTOE1*.

Like certain other Brassicaceae plants, *Arabidopsis* has six AP2 group genes, which is unique because the orthologs of *TOE2, TOE3, SMZ*, and *SNZ* were not found in the other spermatophytes analyzed in this study. Accordingly, the detailed genomic information available for *Arabidopsis* was very helpful for exploring the expansion of the AP2 group in spermatophytes. Alignment of AP2 group genes of *Arabidopsis* revealed conserved regions (Figure 6). Ten exons were identified in the *AtAP2* genomic DNA sequence, nine in *AtTOE1–3* and seven in *AtSMZ* and *AtSNZ*. Unsurprisingly, the exons corresponding to the first EAR, NLS, AP2-R1, and linkage domains exhibited stronger conservation in these six genes, and the conserved sequences of the AP2-R2 domain in *AtAP2, AtTOE1* and *AtTOE3* are also shown in Figure 6. Most notably, a region in intron 5 of *AtTOE2* was very similar to exon 6 of *AtAP2, AtTOE1*, and *AtTOE3*, strongly suggesting that exon 6 was lost in the course of *AtTOE2* evolution. There was also some sequence conservation in introns 1, 3, and 4. Overall, *AtTOE1* and *AtTOE2* were the most closely related of these six genes, and the similarity between *AtSMZ* and *AtSNZ* was highest. The conservation among other genes was mainly in the introns, such as between *AtTOE3* and *AtSMZ* and between *AtTOE2* and *AtSNZ*. In the phylogenetic tree of all AP2 group genes in Brassicaceae (Figure 1 and Image 3), the relationship between clades SMZ and SNZ was paralogous and so were the relationships between the AP2 clade and the TOE3, TOE2, and SMZ/SNZ clades and the AP2 and TOE1 types. These results indicated that gene duplication was an important cause of the expansion of the Brassicaceae AP2 group. Structural changes and rearrangements after gene duplication could have resulted in functional divergence.

### *Arabidopsis* mutant analysis supports evidence for functional divergence after gene expansion of the AP2 group

Mutant studies indicate that *AP2* is involved in the regulation of the stem-cell niche in the shoot meristem (Wurschum et al., 2005), floral development (Jofuku et al., 1994), and seed mass (Ohto et al., 2005), whereas *AtSMZ, AtSNZ, AtTOE1, AtTOE2*, and *AtTOE3* redundantly affect flowering time (Jung et al., 2007, 2014; Yant et al., 2010). We isolated a novel *ap2* allele from an ethyl methanesulfonate mutagenesis screen for abnormal expression patterns of the shoot meristem stem cell marker *pCLV3*:*YFPer* transgenic line. In contrast to wild-type plants, where *pCLV3:YFPer* is expressed in the three stem-cell layers of torpedo-stage embryos (Image 4A), *pCLV3:YFPer* signal was only observed in the epidermal layer of the mutant *ap2* (*2-132*) mutant at levels comparable to wild-type, whereas expression in the subjacent layers was strongly reduced (Image 4B). At the seedling stage, *ap2* (*2-132*) mutants failed to develop a wild-type like shoot meristem. *ap2* (*2-132*) plants also were late flowering with abnormal flower phenotype and abnormal *CLV3* expression in the stem-cell niche of shoots (Image 4A–D,H).

Comparing the genomic and coding sequences of *AtAP2* in the wild type and *2-132*, a single-base exchange in intron 6 of *AtAP2* was identified (Image 4E), which affected pre-mRNA splicing and led to loss of exon 6 of *AtAP2* in *2-132* (Image 4F), and consequently 15 amino acids were lost from the AP2-R2 domain (Image 4G). Remarkably, exon loss also occurred in *AtTOE2*, and this lost exon is homologous with exon 6 of *AtAP2* (Figure 6 and Image 4G). Thus, fluctuation in the number of exons may facilitate divergence in gene function and also may be one of the ways new genes are formed.

## Discussion

### Typical AP2 group genes first appeared in gymnosperms and evolved into AP2 and TOE types through whole-genome and gene duplication in angiosperms

The AP2 domain was previously considered a plant specific core construct of the AP2 family, but it has more recently also been found in cyanobacterium, ciliates, and viruses (Magnani et al., 2004). These newly identified non-plant proteins with an AP2 domain are predicted HNH endonucleases, a kind of homing endonuclease (Chevalier and Stoddard, 2001). An HNH-AP2 homing endonuclease may have been transported into plants via endosymbiosis, horizontal transfer or other lateral gene transfer events. In the process of formation of the AP2 subfamily containing two AP2 domains, tandem duplication is likely to have played a major role (Magnani et al., 2004). A protein containing two AP2 domains has been identified in *Chlamydomonas reinhardtii*, but it does not clustered with the AP2 subfamily, though the amino acid composition of its AP2 domain is very similar to the AP2 group (Shigyo et al., 2006). It is regarded as a sister to the AP2 and ANT groups in terms of phylogenetic relationships (Shigyo et al., 2006). AP2/ERF proteins with two AP2 domains from *Physcomitrella* cluster with the ANT group (Kim et al., 2006). *C. reinhardtii* belongs to the Chlorophyta lineage, which is sister to the Streptophyta lineage (Charophyceae and land plants, Karol et al., 2001). This suggests that the AP2 and EREBP subfamilies diverged before the Chlorophyta lineage diverged from the Streptophyta lineage (Shigyo et al., 2006). In addition, we found no orthologs of AP2 group genes in our database searches of alga, moss and fern. *AP2L* from gymnosperms were identified in searching the orthologs of AP2 group genes, demonstrating the ancestral polyploidy event during the formation of gymnosperms (Jiao et al., 2011). AP2 group genes were also detected in basal angiosperms (*Amborella trichopoda, AmtAP2*; *Gnetum parvifolium, GpTOE1*; *Nymphaea hybrid cultivar, NhTOE1*) and respectively clustered into the AP2 and TOE1 clades. The presence of angiosperm genes in both the AP2- and TOE-type lineages suggests the duplication that gave rise to these two lineages followed the divergence of gymnosperms and angiosperms. The whole-genome duplication in ancestral angiosperms (Jiao et al., 2011) may have led to the AP2 group genes falling into two broad categories: the AP2 and TOE types.

### Gene duplication and motif changes produced new genes in the angiosperm AP2 group

The distribution diagram (Figure 2) of motifs and homeodomains exhibited the evolutionary relationships in the AP2 group. There were ANT orthologs but no AP2 orthologs in alga, moss and fern, which supports the argument that the AP2 group first appeared in gymnosperms. There was no differentiation between the AP2 and TOE types in gymnosperms, though all *AP2Ls* from Pinaceae were divided into two sub-branches by the whole-genome duplication in ancestral gymnosperms, which indicates that the duplication did not lead to the formation of TOE-type genes. The whole-genome duplication in the ancestral angiosperm was the likely basis of AP2 group gene differentiation. In this process, there were more new motifs in AP2-type than TOE-type genes (Figure 2). The homeodomains have evolved little in the AP2 group gene differentiation, especially the NLS and AP2-R1 domains. Most obviously, the first EAR domain transformed from DLNxxP to LxLxL in the TOE type and disappeared in the TOE3 clade. These changes caused AP2 group genes to diverge into the AP2 and TOE types. Our analysis demonstrated there are AP2 and TOE1 orthologs in most angiosperms except Brassicaceae, which contains six AP2 group genes, suggesting that other gene or whole-genome duplication events occurred in the course of evolution. In fact, the extensive complete genome analyses in *Arabidopsis* supports the model that two recent whole-genome duplication events occurred in Brassicaceae and one triplication event occurred in eudicots (Bowers et al., 2003; Tuskan et al., 2006; Lyons et al., 2008; Barker et al., 2009). Interestingly, only in Brassicaceae, new functional AP2 group orthologs appeared. In most angiosperms, polyploidy could simply cause increased gene copy numbers of *AP2* and *TOE1*. Some changes of motifs and homeodomains have taken place in the new AP2 group genes. The AP2 group was different in AP2 domain and motifs than the ANT group (Kim et al., 2006). Like the isolation of AP2 and ANT groups, the new AP2 group genes in Brassicaceae may have been formed by a similar mechanism. For instance, the deletion and amino acid changes mainly occurred in the TOE2 and SMZ/SNZ AP2-R2 domains but TOE3 was formed by the removal of motifs. Compared with TOE2s, SMZs/SNZs evolved two new specific motifs (Figure 2). The analysis of mutant and genomic sequence alignment supports the view that new genes and functional differentiation could be produced by exon changes and genomic sequence rearrangements.

### Different selective pressures drove the evolution of different clades in spermatophyte AP2 group

The selective pressures analysis of all and each clade of the AP2 group genes in spermatophyte suggested that AP2 group genes experienced different evolutionary patterns and each clade encountered various selective pressures, demonstrating that complex selective pressures drove the evolution of the AP2 group. As DNA-binding proteins containing two AP2 domains, AP2 group genes needed to maintain high conservation in the AP2 domains and NLS i.e., to facilitate nuclear translocation and DNA binding. All ω-values were < 1 in the one-by-one comparisons of AP2 group genes, showing a background of purifying selection during evolution and coinciding with the conservation of sequence. However, likelihood values and parameter estimates in PAML demonstrated the presence of positive selection in the evolution of all AP2 group genes, although the positively selected sites were few (< 3% of sites) and the distribution was relatively concentrated (upstream of the NLS and downstream of the second EAR domain). The analysis further showed that the positive selection occurred exclusively in the AP2 clade. Related to the fact that *AP2* was the main functional gene in the AP2 group, this positive selection might have lead to the protein functional changes. There was no positive selection in other clades of the AP2 group other than the AP2 clade. Accordingly, there may have been positive e selection at the time of divergence of the AP2 and TOE types. In the subsequent evolution, every clade experienced differential selective pressures, particularly the AP2 and TOE1 clades. Notably, more amino acid changes accumulated in the TOE1 clade during evolution, which was supported by the pairwise and sliding-window analysis of *dN* and *dS* in all AP2 group genes. As suggested above, different clades of the AP2 group experienced different evolutionary patterns, which might be associated with gene function. AP2 clade genes, as the main functional gene of the AP2 group, required high conservation and probably changed accordingly with angiosperm diversification.

### AP2 clade genes retained ancestor gene function of AP2 group

Phylogenetic analysis demonstrated that all orthologs of AP2 group genes (AP2L) in gymnosperms belong to the AP2 type and are most closely related to the AP2 clade, which suggests that gymnosperms only contain AP2 homologous genes, and that the ancestor of seed plants had AP2-like genes but no TOE1-like genes. In spermatophyte evolution, the AP2 group genes diversified but some functions might be conserved in the most recent common ancestor of extant spermatophytes. In the model plant *Arabidopsis*, the functions of the six AP2 group members (*AtAP2, AtTOE1–3, AtSMZ*, and *AtSNZ*) have been fully studied, and all are repressors of flowering, but only AtAP2 exhibits multiple functions in the development of flowers, fruit, seeds and stem cells (Wurschum et al., 2005; Mathieu et al., 2009; Huijser and Schmid, 2011; Ripoll et al., 2011). The expression patterns of AP2Ls in gymnosperms (*Larix* × *marschlinsii, P. abies, P. thunbergii*) have been examined in previous studies. AP2Ls are expressed in female and male cones, leaves, stems and roots (Vahala et al., 2001; Shigyo and Ito, 2004). AP2L from P. abies shows functional similarities to AtAP2 in floral patterning when overexpressed in *Arabidopsis* (Nilsson et al., 2007) and the overexpression of AtAP2 also affects floral patterning in *Nicotiana benthamiana* (Mlotshwa et al., 2006). AP2Ls are also expressed during somatic embryogenesis in gymnosperms (Guillaumot et al., 2008). Remarkably, there are multiple homologous genes of the AP2 group in gymnosperms and their expression patterns differ from each other, though they belong to theAP2 type together with the AP2 clade in the phylogenetic tree, which implies that there has been a certain degree of functional differentiation in gymnosperm AP2Ls. Having gone through whole-genome duplication in the ancestral angiosperm, AP2 clade proteins in angiosperms likely had similar functions to gymnosperm AP2Ls inheriting from the common ancestor. This model is widely supported by the fact that AP2 clade proteins from *Oryza sativa* (Lee et al., 2007), *Zea mays* (Chuck et al., 2007), *Hordeum vulgare* (Nair et al., 2010), *Solanum lycopersicon* (Karlova et al., 2011), *Solanum tuberosum* (Martin et al., 2009), *Actinidia deliciosa* (Varkonyi-Gasic et al., 2012), *Petunia hybrida* (Maes et al., 1999, 2001), and *Crocus sativus* (Tsaftaris et al., 2012) have been linked to AP2Ls and they function in floral organ identity and development, fruit development, lodicules development, branching and tuberization. To meet the complex requirements of biological and functional diversity, AP2 clades genes have been constantly evolving by structural changes and adaptive evolution, which is in good agreement with our analysis of structural (motifs and homeodomains) alignment and selective pressure.

### Gene numberic expansion of AP2 group producted new genes with similar functions

With gene or whole-genome duplications in spermatophyte evolution, there was expansion of the number of AP2 group genes, especially in Brassicaceae. The phylogenetic tree of the Brassicaceae AP2 group reflects the evolutionary relationship: The TOE3 clade is a sister to the AP2 clade belonging to the AP2 type; the TOE2 and SMZ/SNZ clades are paralogous and cluster together with the TOE1 clade in the TOE type. The analysis results have gained support from the functional research of AP2 group proteins in *Arabidopsis*. AP2 group proteins from *Arabidopsis* are control factors of flowering. AP2-type proteins (AtAP2 and AtTOE3) participate in floral organ identity and development (Aukerman and Sakai, 2003; Chen, 2004; Jung et al., 2014), but TOE-type proteins (AtTOE1 and AtTOE2) are involved in flowering control and developmental phases of plant (Huijser and Schmid, 2011). The single mutant and overexpression of *AtTOE3* showed no visible phenotypic effects, while the expression pattern was different from other AP2 group genes in *Arabidopsis* (Jung et al., 2007; Yant et al., 2010). Recent research has shown that overexpression of an miR172-resistant *AtTOE3* can control floral organ identity and flowering time when the miR172 target site is mutated (Jung et al., 2014). AtTOE3 binds to the second intron of *AGAMOUS* (*AtAG*) and represses its expression like AtAP2 (Yant et al., 2010; Jung et al., 2014), which means that the function of AtTOE3 is similar to AtAP2, but is strongly constrained by *miR172*. Overexpression of *AtSMZ, AtSNZ, AtTOE1*, or *AtTOE2* causes late flowering (Aukerman and Sakai, 2003; Chen, 2004; Jung et al., 2007) and quadruple (*smz snz toe1 toe2*) and sextuple (*ap2 toe3 smz snz toe1 toe2*) mutants flower earlier than any single or double mutant (Jung et al., 2007; Mathieu et al., 2009; Yant et al., 2010), which also indicates that the function of TOE-type genes is similar in *Arabidopsis*. The functional studies of the AP2 group proteins in *Arabidopsis* supports the model that orthologs formed by gene or whole-genome duplications could be transformed into new genes by changes in motifs and homeodomains.

### *AP2* function to maintain the stem cell niche was conservative function in spermatophyte

In *Arabidopsis*, the marker genes *WUS* and *CLV3* of shoot meristem stem-cell niche are regulated by AP2 and TOE3, and TOE1 does not act redundantly with AP2 in stem-cell maintenance (Wurschum et al., 2005). Likewise, the expression of *WUS* and *CLV3* is changed in *Arabidopsis* by overexpressing *AP2L* of *P. abies* (Nilsson et al., 2007). Overexpression of *AtAP2* in *N. benthamiana* also causes expression changes of *NbWUS* (Mlotshwa et al., 2006). Accordingly, the function of maintaining the stem-cell niche was conserved in the spermatophyte AP2 and AP2L clades. *AP2Ls* from *Larix* × *marschlinsii* are expressed during somatic embryogenesis and germination (Guillaumot et al., 2008), which also provides support for this view. Mutant analysis showed that changes in AP2-R1 and R2 lead to loss of stem-cell maintenance. *AtTOE3* and *AtTOE1* do not function in stem-cell maintenance, which indicates that the changes in motifs and homeodomains could also cause the loss of function in stem-cell maintenance.

The spermatophyte AP2 group contains many orthologs because of gene or whole-genome duplications. These orthologs may have evolved different functions, which means that the AP2 group may be a very valuable research area for examining new gene formation. The problems in AP2 group research focus on two aspects: evolution and functional differentiation. In gymnosperm, there was no differentiation into AP2 type and TOE type. In angiosperm, AP2 group genes were divided into two types and finished the functional differentiation. But more gene and genome data was needed to support the conclusion, especially gymnosperm. There are only two members (*AP2s* and *TOE1s*) in angiosperm AP2 group expect Brassicaceae. Current data suggested *TOE2s, TOE3s, SMZs*, and *SNZs* only exist in Brassicaceae, which means gene expansion and functional differentiation occurred during the formation of Brassicaceae but the detailed process was still unknown. *AP2s* are the the main function genes in AP2 group. After the formation of *TOE1s*, the function of flower time was only preserved. In Brassicaceae, *SMZs* and *SNZs* that belong to the same type with *TOE1s* own some new gene function. But so far, that still needs to be further studied. The correspondence among expression patterns, function and phylogenetic relatedness requires further study. The functionally similar genes in this study suggest functional diversification within the AP2 group. Comprehensive studies of expression and function and intensive phylogenetic characterization of the AP2 group genes will give us a clearer indication of the roles these genes play in the developmental processes of different species as well as the function of AP2 group genes in the evolutionary history of spermatophytes.

## Materials and methods

### Sequence data

We retrieved the nucleotide and amino acid sequences for the *Arabidopsis* AP2 group from the Arabidopsis Information Resource database (www.arabidopsis.org). A BLASTP search was then performed using the AtAP2, AtTOE1–3, AtSMZ, and AtSNZ sequences as the query to retrieve AP2 group gene sequences from the NCBI (www.ncbi.nlm.nih.gov) and Phytozome databases (www.phytozome.org). The identified sequences were from the 56 species of spermatophytes (Data sheet 1). All selected AP2 group amino acid sequences contain six or seven conserved domains (Image 1). Prior to the phylogenetic analysis of these AP2 group gene protein sequences with *Arabidopsis* AP2 group, we ensured that all the AP2 group sequences clustered together as well as with the AtAP2 group. For our analysis, we selected the most typical AP2 group genes from a large number of paralogs but retained all query results of gymnosperms (Data sheet 1). The genes that fell outside the AP2 group were not analyzed.

### Phylogenetic analysis and tree construction

After deletion of identical sequences, only 105 sequences were used for phylogenetic analysis (Data sheet 1). They were aligned together using CLUSTAL 1.83. The phylogenetic tree of AP2 group genes was obtained by using ML (maximum likelihood) (MEGA 6.0) methods, and the reliability of the trees was evaluated by the bootstrap method with 1000 replications. The *dN*/*dS* value was used to detect positive selection.

### Identification of sequence motifs

To identify motifs shared among related proteins within the AP2 group gene, we used the MEME motif search tool was used with its default settings. The maximum number of possible motifs was set to 35, and the maximum width was 300. Identified motifs were annotated using SMART (http://smart.embl-heidelberg.de/) and Pfam (http://pfam.sanger.ac.uk/).

### Analysis of adaptive evolution and identification of selective pressures

The program Codeml implemented in the PAML 4.0 software package was used to investigate the adaptive evolution of AP2 group protein-coding sequences. A total of 105 aligned AP2 group genes sequences, isolated from the different clades, were selected to test whether they were under purifying selection. Six models of codon substitution, M0 (one-ratio), M1a (Nearly Neutral), M2a (Positive Selection), M3 (discrete), M7 (beta), and M8 (beta & ω) were used in the analysis. M0 assumes that all sites have the same ω ratio. M1a assumes two classes of sites in proteins in proportions p0 and p1 (1–p0) with 0 < ω_0_ < 1 (purifying selection) and ω_1_ = 1 (neutral sites). M2a adds a proportion (p2) to account for a class of sites where ω_2_ is estimated from the data and can be > 1. M3 uses a general discrete distribution with three site classes, with the proportions (p0, p1, and p2) and the ω ratios (ω_0_, ω_1_, and ω_2_) estimated from the data. M7 assumes a beta distribution (p, q) for 10 different ω ratios in the interval (0, 1). M8 adds an extra class of sites with positive selection (ω > 1) to the beta (M7) model. Therefore, the null models M0, M1a and M7 fix the ω ratios between 0 and 1, and do not allow the presence of positively selected sites. The alternative models M2a, M3, and M8 account for positive selection by using parameters, which estimate ω greater than 1, and allow for the variable ω along codon sequence.

The likelihood ratio test (LRT) was performed to detect the presence of positively selected sites by comparing the models that do not allow for positive selection with the models that allow for positive selection. The LRT was performed by taking twice the difference in log likelihood between nested models and testing for significance using the χ2 distribution with the degrees of freedom equivalent to the difference in the number of parameters between models. If the LRT yields a statistically significant result, then positive selection is inferred. In the present study, three LRTs (M0 vs. M3, M1a vs. M2a, and M7 vs. M8) were used to detect positive selection. The Bayes empirical Bayes (BEB) approach implemented in M2a and M8 was used to determine the positively selected sites by calculating the posterior probabilities (p) of ω classes for each site. The sites with high posterior probabilities (*p* > 0.95) coming from the class with ω > 1 were believed to be under positive selection.

### Plant growth, mutant lines, and mapping

All plants were in the Landsberg erecta (Ler) ecotype, which was also used as the wild-type control. The *2-132* mutant was identified in the M2 generation of ethylmethanesulfonate-mutagenized *pCLV3:YFPer* plants. *ap2-1* and *ap2-2* mutants have been described.

The *2-132* mutation was mapped using plants with a wild-type-like phenotype in the F2 generation from a cross of an *2-132/*+ plant with the Columbia ecotype. The initial mapping was done using CAPS markers from The Arabidopsis Information Resource (http://www.arabidopsis.org). The dCAPS markers used for fine mapping of the *2-132* mutation were generated based on the information available from CEREON.

## Author contributions

PW, JS, and JC planned and designed the research. PW, TC, and Meiping Li performed experiments. Mengzhu Lu, TL conducted experiments. PW, GL, YL, and JC collected and analyzed data. PW wrote the manuscript.

## Funding

This work was supported by National High Technology Research and Development Program of China (863 Program, 2013AA102705), Specialized National Basic Research Program of China (973 Program, 2012CB114504), Natural Science Foundation of Jiangsu University grant 13KJA220001, Talent project by the Ministry of Science and Technology, Co-Innovation Center for Sustainable Forestry in Southern China, Nanjing Forestry University, and Priority Academic Program Development of Jiangsu Higher Education Institutions.

### Conflict of interest statement

The authors declare that the research was conducted in the absence of any commercial or financial relationships that could be construed as a potential conflict of interest.

## Supplementary material

Image 1 **Conservative Domains Distributions of AP2 Group and the Consensus Amino Acid Sequence of AP2-R2 Domain in Brassicaceae**. Different colors represent different conservative domains (EAR domain, yellow; purple, NLS domain; aqua, AP2-R1 domain; gold, linkage domain; red and green, AP2-R2 domain; darkcyan, miRNA172 target site). The amino acid composition of AP2-R2 domain in different AP2 group genes is poor conservative, especially in TOE2s, SMZs, and SNZs.

Image 2 **Detailed Phylogenetic Trees of five clade in Figure 1**. The different colorst represent different clades (red, AP2 clades; green, TOE1 clade; olive, TOE2 clade; purple, TOE3 clade; blue, SMZ/SNZ clade). The species and accession numbers are listed in Data sheet 1. The abbreviations used are as follows: *Ad, Actinidia deliciosa*; *Amt, Amborella trichopoda*; *Bp, Betula platyphylla*; *Al, Arabidopsis lyrata*; *At, Arabidopsis thaliana*; *Aa, Arabis alpine*; *Bn, Brassica napus*; *Br, Brassica rapa*; *Cr, Capsella rubella*; *La, Lepidium appelianum*; *Th, Thellungiella halophile*; *Cp, Carica papaya*; *Jc, Jatropha curcas*; *Rc, Ricinus communis*; *Me, Manihot esculenta*; *Ca, Cicer arietinum*; *Gm, Glycine max*; *Lj, Lotus japonicas*; *Mt, Medicago truncatula*; *Pv, Phaseolus vulgaris*; *Ps, Pisum sativum*; *Gp, Gnetum parvifolium*; *Gr, Gossypium raimondii*; *Eg, Eucalyptus grandis*; *Nh, Nymphaea hybrid cultivar*; *Aes, Aegilops speltoides*; *Aet, Aegilops tauschii*; *Bd, Brachypodium distachyon*; *Hv, Hordeum vulgare*; *Os, Oryza sativa*; *Si, Setaria italic*; *Sb, Sorghum bicolor*; *Tt, Triticum turanicum*; *Zm, Zea mays*; *Fv, Fragaria vesca*; *Md, Malus* × *domestica*; *Pp, Prunus persica*; *Cc, Citrus clementina*; *Cs, Citrus sinensis*; *Ct, Citrus trifoliate*; *Pt, Populus trichocarpa*; *Anm, Antirrhinum majus*; *Mg, Mimulus guttatus*; *Ph, Petunia* × *hybrid*; *Sl, Solanum lycopersicum*; *St, Solanum tuberosum*; *Tc, Theobroma cacao*; *Cas, Camellia sinensis*; *Vv, Vitis vinifera*.

Image 3 **Phylogenetic Analysis of AP2 Group Protein in Brassicaceae (simplified phylogenetic tree)**. The ML tree was constructed based on the whole protein sequences of spermatophyte AP2 Group gene using MEGA6.0 with 1000 bootstrap replications and Jones-Taylor-Thornton (JTT) + Gamma Distributed model (Discrete Gamma Categories = 5).

Image 4 **Phenotype, Site of Mutation and Mechanism of Mutation of ***2-132***. (A,B)** Fluorescence microscope of pCLV3:YFPer in wild-type **(A)** and *2-132* **(B)** homozygous mutant torpedo stage embryos. The yellow fuorescent protein indicate the location of the embryonic stem cell niche. In 100 self-crossed progeny embryos of *2-132* heterozygote, the number of abnormal and normal yellow fuorescent in embryonic stem cell niche was 29: 71, which was no significant difference with 3:1 by χ^2^-test. **(C,D)** The phenotype of flower in in wild-type **(C)** and *2-132* **(D)** homozygous mutant. The sepals of *2-132* **(D)** homozygous mutant transform into leaves morphologically and the petals are like sepals. E Genomic organization of AP2. The mutant sites of the *2-132, l28, ap2-1, ap2-2*, and *ap2-7* mutations are shown. The exon sequences of the two AP2 domains are marked (aqua, AP2-R1 domain; red, AP2-R2 domain). The point mutation in the genomic sequence of *2-132* is highlighted. **(F,G)** The sequencing results of *AP2* (genomic DNA and mRNA) from wild-type and *2-132* homozygous mutant. The sequences of mRNA show there are 45 bases deletion in *AP2* of *2-132* homozygous mutant which happens to be the sixth exon of wild-type *AP2*. In the *AtTOE2* of wild-type, this exon also does not exist. **(H)** The phenotype of flowering and height growth (centimeter) of wild-type and *2-132* homozygous mutant. The number of rosette leaves in *2-132* **(B)** homozygous mutant during flowering time is more than wild-type but the height growth is less.

Data Sheet 1 **The List of AP2 Group Genes in this Article**.

Data Sheet 2 **Branch Model Test for Each Clade Genes**.

Data Sheet 3 **Likelihood Values and Parameter Estimates for ***AP2s, TOE1s, TOE3s, TOE2s***, and ***SMZSNZs*** in Brassicaceae**.
