# Supplementary figures and images for: Expansion and Functional Divergence of AP2 Group Genes in Spermatophytes Determined by Molecular Evolution and Arabidopsis Mutant Analysis
Source: Front Plant Sci. 2016 Sep 20;7:1383. doi: 10.3389/fpls.2016.01383 (PMC5029118; doi:10.3389/fpls.2016.01383)

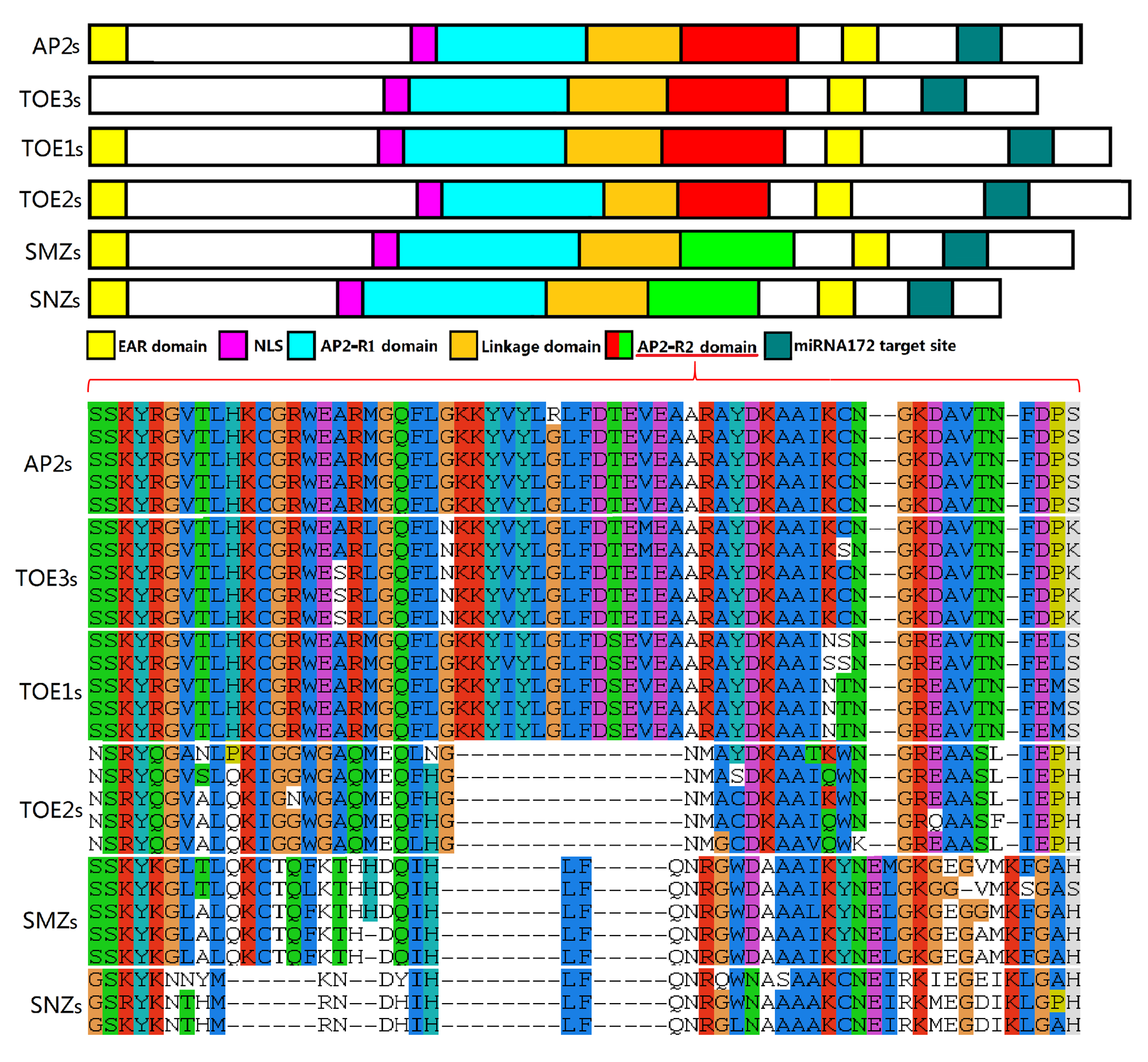

Supplement: Image 1 — Conservative Domains Distributions of AP2 Group and the Consensus Amino Acid Sequence of AP2-R2 Domain in Brassicaceae. Different colors represent different conservative domains (EAR domain, yellow; purple, NLS domain; aqua, AP2-R1 domain; gold, linkage domain; red and green, AP2-R2 domain; darkcyan, miRNA172 target site). The amino acid composition of AP2-R2 domain in different AP2 group genes is poor conservative, especially in TOE2s, SMZs, and SNZs. [file Image1.TIF]

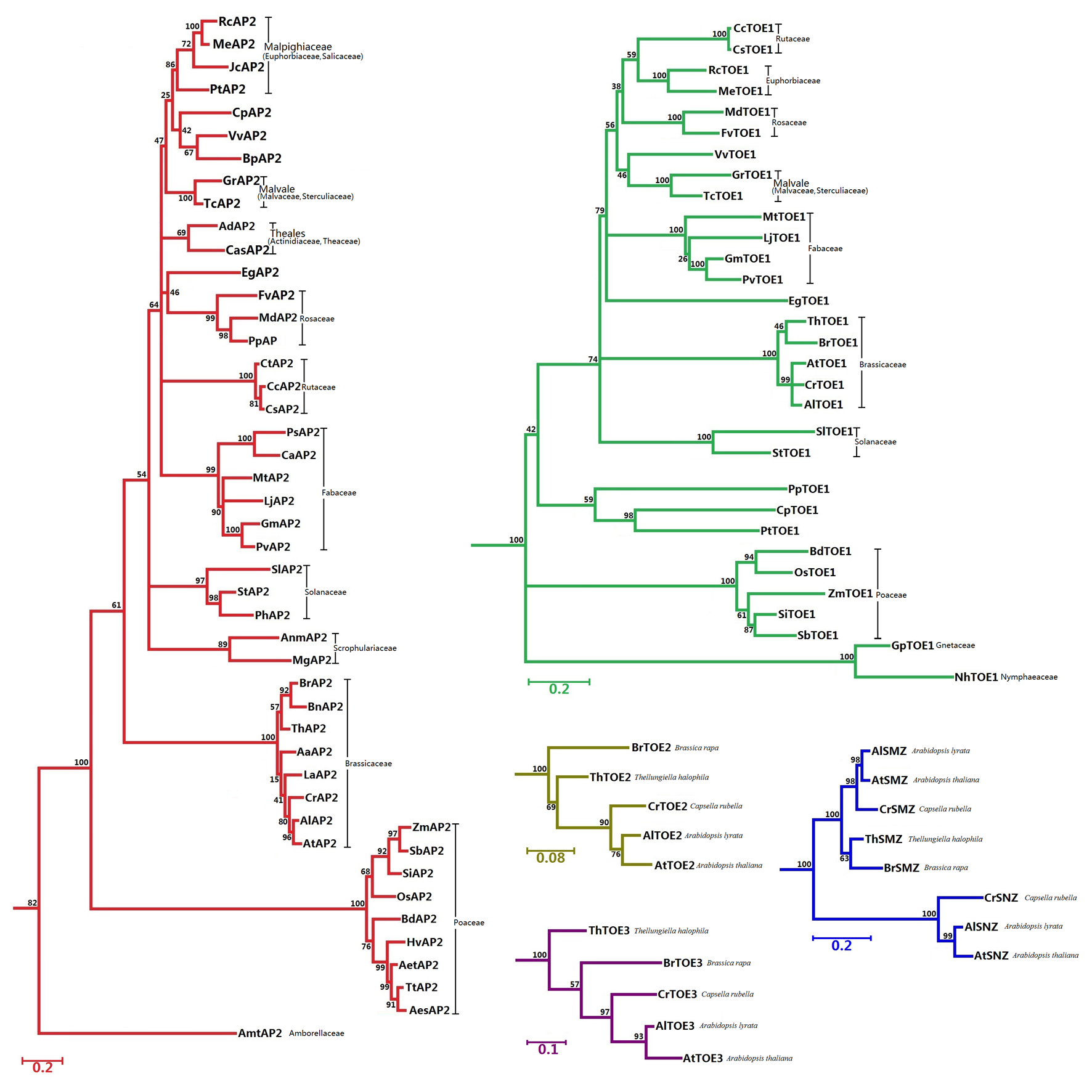

Supplement: Image 2 — Detailed Phylogenetic Trees of five clade in Figure 1. The different colorst represent different clades (red, AP2 clades; green, TOE1 clade; olive, TOE2 clade; purple, TOE3 clade; blue, SMZ/SNZ clade). The species and accession numbers are listed in Data sheet 1. The abbreviations used are as follows: Ad, Actinidia deliciosa; Amt, Amborella trichopoda; Bp, Betula platyphylla; Al, Arabidopsis lyrata; At, Arabidopsis thaliana; Aa, Arabis alpine; Bn, Brassica napus; Br, Brassica rapa; Cr, Capsella rubella; La, Lepidium appelianum; Th, Thellungiella halophile; Cp, Carica papaya; Jc, Jatropha curcas; Rc, Ricinus communis; Me, Manihot esculenta; Ca, Cicer arietinum; Gm, Glycine max; Lj, Lotus japonicas; Mt, Medicago truncatula; Pv, Phaseolus vulgaris; Ps, Pisum sativum; Gp, Gnetum parvifolium; Gr, Gossypium raimondii; Eg, Eucalyptus grandis; Nh, Nymphaea hybrid cultivar; Aes, Aegilops speltoides; Aet, Aegilops tauschii; Bd, Brachypodium distachyon; Hv, Hordeum vulgare; Os, Oryza sativa; Si, Setaria italic; Sb, Sorghum bicolor; Tt, Triticum turanicum; Zm, Zea mays; Fv, Fragaria vesca; Md, Malus × domestica; Pp, Prunus persica; Cc, Citrus clementina; Cs, Citrus sinensis; Ct, Citrus trifoliate; Pt, Populus trichocarpa; Anm, Antirrhinum majus; Mg, Mimulus guttatus; Ph, Petunia × hybrid; Sl, Solanum lycopersicum; St, Solanum tuberosum; Tc, Theobroma cacao; Cas, Camellia sinensis; Vv, Vitis vinifera. [file Image2.TIF]

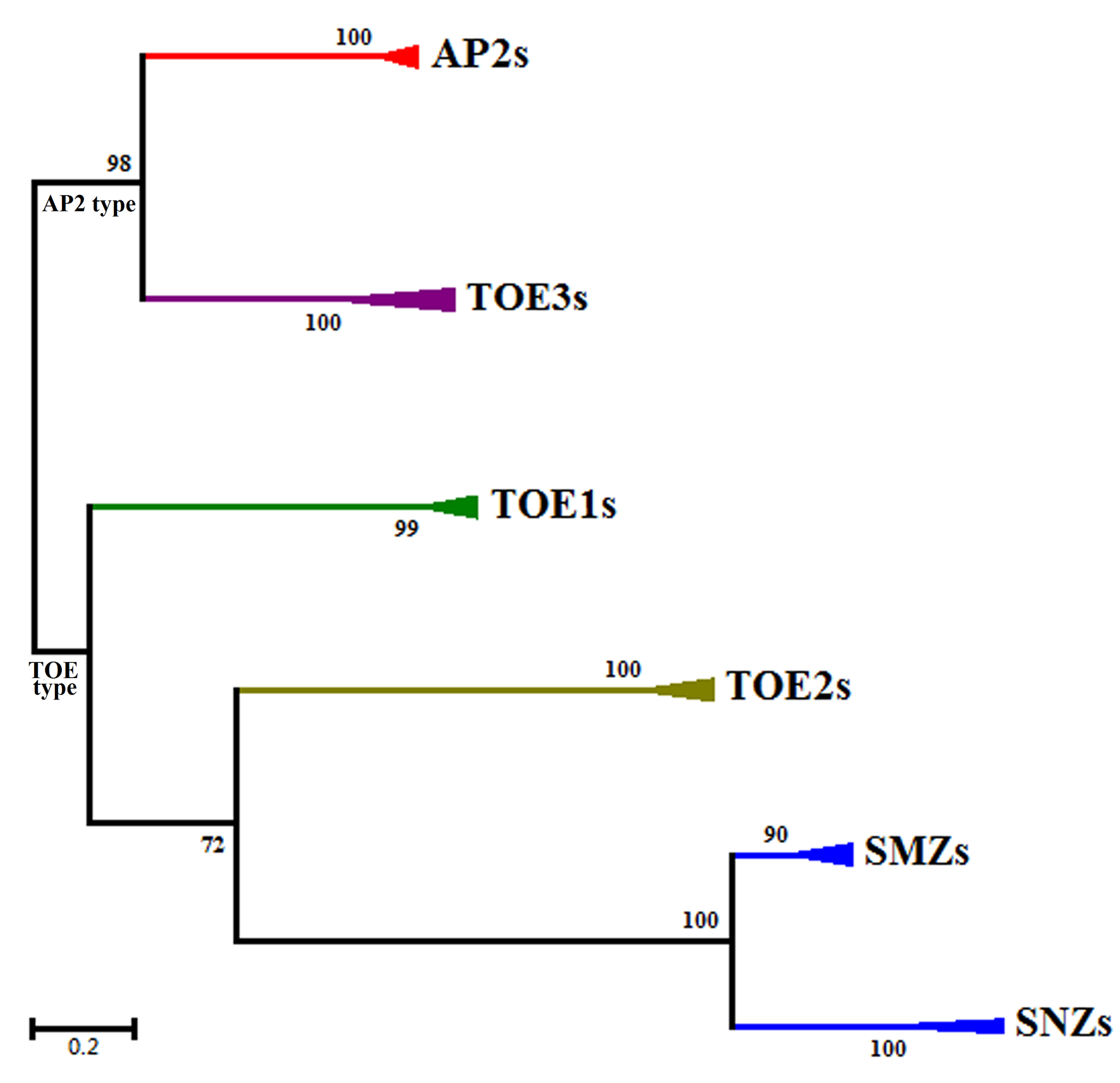

Supplement: Image 3 — Phylogenetic Analysis of AP2 Group Protein in Brassicaceae (simplified phylogenetic tree). The ML tree was constructed based on the whole protein sequences of spermatophyte AP2 Group gene using MEGA6.0 with 1000 bootstrap replications and Jones-Taylor-Thornton (JTT) + Gamma Distributed model (Discrete Gamma Categories = 5). [file Image3.TIF]

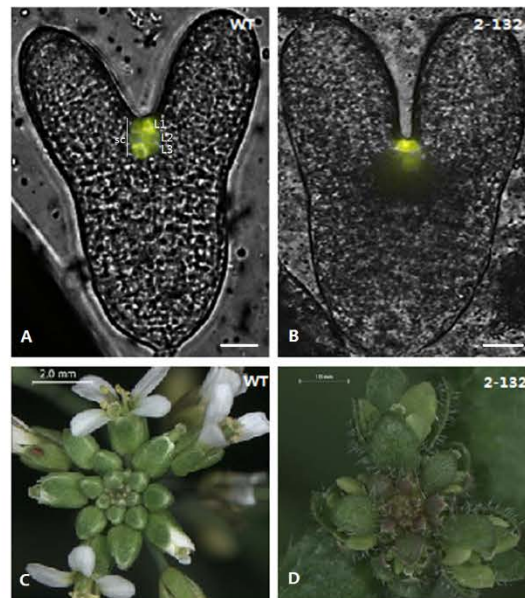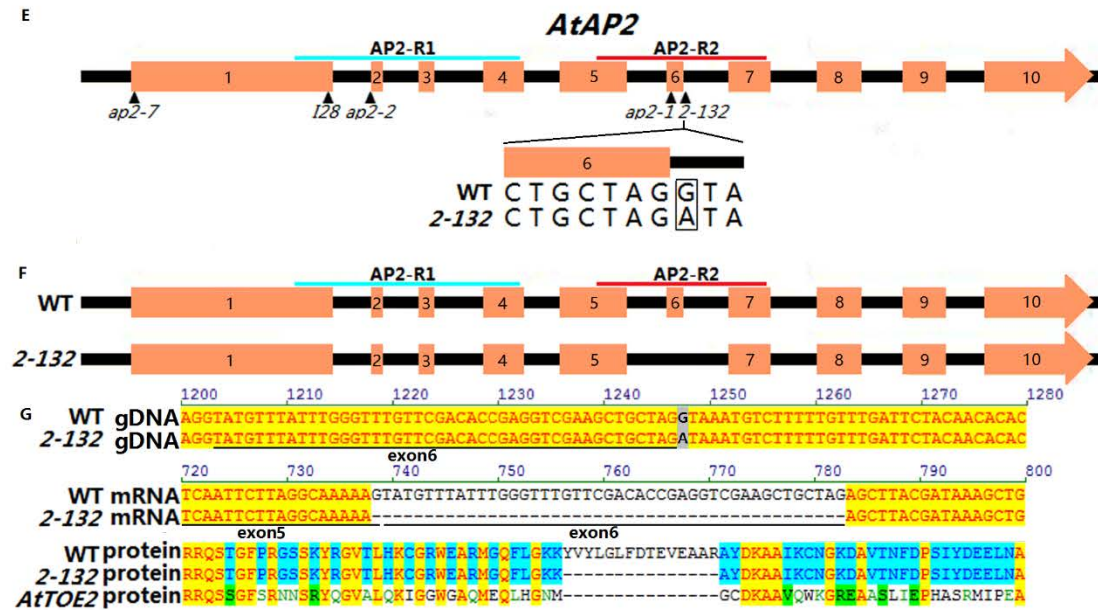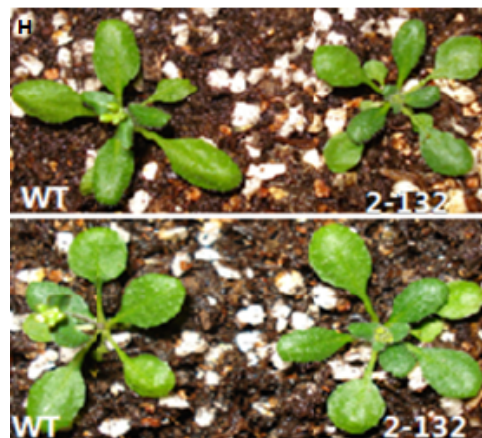

| Genotype | <i>n</i> | Rosette Leaves | Plant Height |
|----------|----------|----------------|--------------|
| WT       | 47       | 6.6 ± 0.9      | 27.1 ± 2.0   |
| 2-132    | 43       | 8.1 ± 1.0      | 24.1 ± 1.9   |

Supplement: Image 4 — Phenotype, Site of Mutation and Mechanism of Mutation of 2-132. (A,B) Fluorescence microscope of pCLV3:YFPer in wild-type (A) and 2-132 (B) homozygous mutant torpedo stage embryos. The yellow fuorescent protein indicate the location of the embryonic stem cell niche. In 100 self-crossed progeny embryos of 2-132 heterozygote, the number of abnormal and normal yellow fuorescent in embryonic stem cell niche was 29: 71, which was no significant difference with 3:1 by χ2-test. (C,D) The phenotype of flower in in wild-type (C) and 2-132 (D) homozygous mutant. The sepals of 2-132 (D) homozygous mutant transform into leaves morphologically and the petals are like sepals. E Genomic organization of AP2. The mutant sites of the 2-132, l28, ap2-1, ap2-2, and ap2-7 mutations are shown. The exon sequences of the two AP2 domains are marked (aqua, AP2-R1 domain; red, AP2-R2 domain). The point mutation in the genomic sequence of 2-132 is highlighted. (F,G) The sequencing results of AP2 (genomic DNA and mRNA) from wild-type and 2-132 homozygous mutant. The sequences of mRNA show there are 45 bases deletion in AP2 of 2-132 homozygous mutant which happens to be the sixth exon of wild-type AP2. In the AtTOE2 of wild-type, this exon also does not exist. (H) The phenotype of flowering and height growth (centimeter) of wild-type and 2-132 homozygous mutant. The number of rosette leaves in 2-132 (B) homozygous mutant during flowering time is more than wild-type but the height growth is less. [file Image4.PDF]
